# Glutamatergic and dopaminergic modulation of cortico-striatal circuits probed by dynamic calcium imaging of networks reconstructed in microfluidic chips

**DOI:** 10.1038/s41598-018-35802-9

**Published:** 2018-11-29

**Authors:** Benjamin Lassus, Jérémie Naudé, Philippe Faure, Denis Guedin, Ysander Von Boxberg, Clotilde Mannoury la Cour, Mark J. Millan, Jean-Michel Peyrin

**Affiliations:** 1 CNRS UMR 8256, Biological Adaptation and Ageing, Paris, 75005 France; 2 CNRS UMR 8246, Neurosciences, Paris, 75005 France; 3 Sorbonne Universités, UPMC, Institut de Biologie Paris-Seine, 75005 Paris, France; 4 Servier Biotechnology, Chemogenetic Laboratory @ ICM Brain & Spine Institue, Pitié-Salpétrière Hospital, 52 boulevard Vincent Auriol, 75013 Paris, France; 5 Centre for Therapeutic Innovation in Neuropsychiatry, Institut de Recherches Servier, Centre de Recherches de Croissy, 78290 Croissy-sur-Seine, France

## Abstract

Although the prefrontal cortex and basal ganglia are functionally interconnected by parallel loops, cellular substrates underlying their interaction remain poorly understood. One novel approach for addressing this issue is microfluidics, a methodology which recapitulates several intrinsic and synaptic properties of cortico-subcortical networks. We developed a microfluidic device where cortical neurons projected onto striatal neurons in a separate compartment. We exploited real-time (low-resolution/high-output) calcium imaging to register network dynamics and characterize the response to glutamatergic and dopaminergic agents. Reconstructed cortico-striatal networks revealed the progressive appearance of cortical VGLUT1 clusters on striatal dendrites, correlating with the emergence of spontaneous and synchronous glutamatergic responses of striatal neurons to concurrent cortical stimulation. Striatal exposure to the NMDA receptor GluN2A subunit antagonist TCN201 did not affect network rhythm, whereas the GluN2B subunit antagonist RO256981 significantly decreased striatal activity. Dopamine application or the D2/D3 receptor agonist, quinpirole, decreased cortico-striatal synchrony whereas the D1 receptor agonist, SKF38393, was ineffective. These data show that cortico-striatal networks reconstructed in a microfluidic environment are synchronized and present characteristics close to those of their *in situ* counterparts. They should prove instructive for deciphering the molecular substrates of CNS disorders and evaluating the actions of novel therapeutic agents.

## Introduction

The basal ganglia loop is a group of subcortical nuclei comprising the striatum (caudate nucleus, putamen), nucleus accumbens, internal and external globus pallidus, substantia nigra and thalamic nuclei. Together these structures are involved in a variety of processes such as motor control, decision-making, reward processing and learning. Striatal medium spiny neurons (MSNs) represent the main neuronal type in the striatum. MSNs mainly receive two distinct types of afferents on their dendritic spines, a glutamatergic one from the cortex and a dopaminergic (DA) one from the substantia nigra/ventral tegmental area^1,2^. Glutamate is transported by vesicular glutamate transporters (VGLUTs) into synaptic vesicles to reach synaptic compartments^3^. Once released in the synaptic cleft, glutamate binds to two classes of receptors, ionotropic (channels) receptors such as NMDA (N-methyl-D-aspartate), AMPA and kaïnate, and metabotropic receptors. NMDA channels are composed of 2 GluN1 and two GluN2 (A to D) or GluN3 (A or B) subunits (for review see^4,5^). Metabotropic receptors (mGluR), coupled to G-proteins, include 3 groups, mGlurR I to III, activating PLC for group I and inhibiting adenylyl-cyclase for groups II and III. Dopamine likewise modulates glutamatergic transmission, dendritic integration, and MSN intrinsic excitability. Specific classes of DA receptors, involving different signalization pathways, may exert differential effects on voltage- and ligand-gated channels. Indeed, each class of DA receptor activates distinct G-protein related pathways which have a role in neuronal plasticity. D1-like receptors (D1R & D5R) stimulate G_s_ and G_olf_ proteins leading to recruitment of adenylyl-cyclase, increases in levels of cAMP and activation of PKA. This kinase has a wide range of cellular targets, including voltage-gated ion channels and synaptic populations of glutamatergic and other classes of receptor. Conversely, D2-like receptors (D2R, D2R and D4R) stimulate G_i_/G_o_ proteins to inhibit adenylyl cyclase *via* Gα subunits and they also activate PLC through Gβ/γ^6^. Through its spatially-extended release and its effects on synaptic and intrinsic properties, DA affects the coordination of MSN activities, and thus modulates the synchrony and oscillatory behavior of striatal circuits^7^.

Outputs from the basal ganglia loop are classically described as two parallel (direct and indirect) pathways. MSNs mainly express D1R (MSN from the “direct” pathway, dMSN) or D2R (“indirect” pathway, iMSN). These subpopulations are thought to underlie contrasting functions in motor control and learning. Along the same lines, it is believed that DA exerts opposite effects on these neurons, and accordingly on network dynamics: whereas D2R activation in iMSNs modifies the up-state transition and diminishes up-state spiking, exactly the opposite effect is observed after dMSN D1R activation^8,9^. The balance between DA and glutamate activity is critical for the control of cortico-striatal rhythms, and its disruption can lead to motor deficits and/or cognitive dysfunctions.

Collectively, these observations raise the question of the influence of distinct classes of DA receptors on synchrony and oscillatory activity. Studying the influence of dopaminergic inputs on collective striatal dynamics is however complicated by the existence of multiple loops (cortico-striatal, thalamo-striatal and thalamo-cortical). Due to their relative inaccessibility, the majority of studies have been performed in brain slices, but this has limitations due to axotomy and cell death. Further, carrying out a sufficient amount of *in-vivo* recordings (multiple single-unit recordings in distinct cortico-striatal structures) is technically difficult and time-consuming^10,11^.

Herein, we adopted the novel approach of microfluidic biotechnology to decipher the operations and modulations of cortico-striatal pathways. Previous work in this laboratory has shown that building oriented neuronal networks using microfluidic devices is feasible^12^. The goals of the present study were the following. *First*, to establish and validate a microfluidic device for probing cortico-striatal networks. *Second*, to devise statistically robust methods for evaluating the network dynamics through low-resolution/high-output calcium imaging, hence allowing analyses of neuronal frequency, rhythms, and synchrony of the reconstructed networks. *Third*, using this method to characterize the influence of glutamatergic inputs on striatal rhythms. *Fourth*, to likewise explore the modulation of cortico-striatal rhythms by dopamine D2/D3 vs D1 receptors.

## Results

### Cortical fibers trigger progressive maturation of striatal neurons

**Figure 1:**
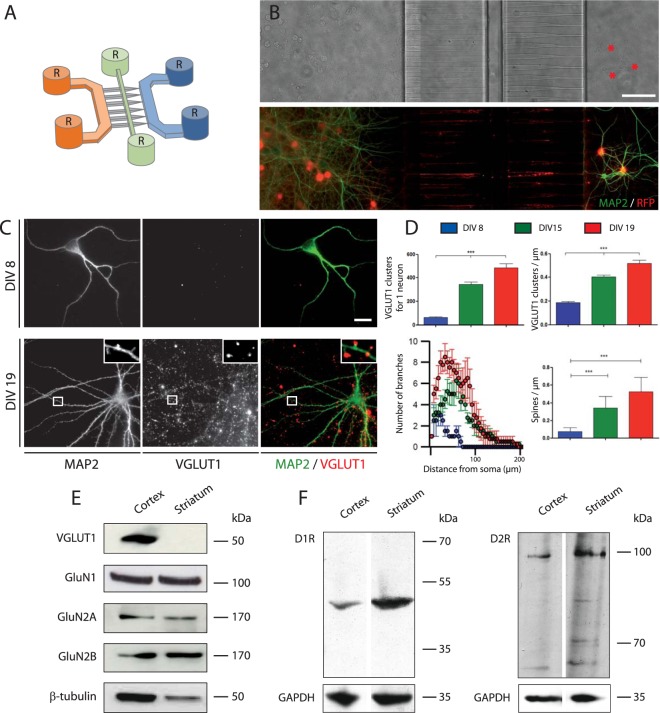
Microfluidic device for the reconstruction of a neuroanatomical pathway, the cortico-striatal network. (**A**) Microfluidic neuronal culture devices are composed of two separate cell culture chambers (orange and blue) linked by a series of asymmetrical, one-way micro-channels (grey) intercepted by a central narrow channel (green). Each chamber is individually perfused by two reservoirs (black R) allowing independent media perfusion between culture chambers. (**B**) Phase contrast image (Fig. 1B top) of a reconstructed oriented cortico-striatal network in the microfluidic device. Red stars indicate striatal neurons. Immunofluorescence of cortico-striatal networks (Fig. 1B bottom). Cortical and striatal neurons are transduced with an RFP-encoding viral vector (RFP red) to visualize axons, and dendritic arborization is labeled with MAP-2 staining (Green). (**C**) Immunofluorescence images of striatal chamber receiving cortical axons at DIV 8 and DIV 19. Notice the pre-synaptic clustering of cortical VGLUT1 on striatal arborization (MAP-2) and the spine formation at DIV19 (insert). (**D**) Assessment of network differentiation with quantification of the number of VGLUT1 clusters, VGLUT1 density (VGLUT1 clusters/µm), dendritic complexity (Sholl analysis) and dendritic spine density (spines/µm), following culture time. Quantification of neuronal development was done on 3 different neuronal cultures. For each culture, quantification was performed on 3 different networks for each time point with a minimum of 10 striatal neurons per network. For each neuron, synaptic density, dendritic complexity and spine density were determined. For spine density the quantification of the number of spines on 100 micrometer crop of dendrites for each neuron were performed. To analyze the results, one-way ANOVA was performed followed by a Bonferroni post-hoc test for synaptogenesis, spinogenesis and dendritic complexity (***P < 0,001). (**E**) Western blot analysis of major proteins linked to ionotropic glutamatergic transmission from DIV19 cortico-striatal network (VGLUT1, GluN1, GluN2A, and GluN2B, whole scan of the western blot films available in supplementary data). (**F**) Western blots of dopamine D1 and D2 receptors from DIV18 cortical neurons and DIV20 striatal neurons, whole scan of the western blot films available in supplementary data.

In order to assess the influence of cortical axons on striatal neuron differentiation, a time-dependent study of cortico-striatal connectivity was carried out. Cortical and striatal neurons were seeded in the left (Fig. 1A Orange) and right (Fig. 1B Blue) chambers respectively. While cortical axons gradually projected through the micro-channels area (Fig. 1A grey) striatal neurons could not project their axons onto cortical neurons owing to the diodes^12^. After 19 DIV, oriented and connected cortico-striatal networks were routinely obtained in microfluidic chips (Fig. 1B). Each microfluidic compartment was fluidically accessible through perfusion and transduction of reconstructed networks with RFP-encoding lentiviral particles allowed visualization of networks subcomponents (Fig. 1B). As shown in Fig. 1C, sequential analysis of network establishment shows that striatal neurons progressively matured upon cortical innervation. The characterization of the striatal MAP-2 cytoskeleton, using Sholl analysis, showed a progressive increase in striatal dendritic complexity upon innervation of striatal neurons by cortical axons from DIV8 to DIV 19 (Fig. 1C). VGLUT1 was chosen as a presynaptic reporter of cortical pyramidal neurons because it is absent in GABAergic striatal neurons^3^. The connection of cortical fibers to striatal neurons induced the progressive formation of cortico-striatal VGLUT1 clusters at striatal dendritic spines (Fig. 1D). At DIV19, the network appeared to be fully established with a stable number of cortical VGLUT1 clusters, a maximal clustering of cortical VGLUT1 on the striatal spines and a progressive increase of the dendritic complexity of striatal neurons (Fig. 1C,D). Expression of VGLUT1, NMDA GluN1, GluN2A and GluN2B receptors in the cortical and striatal cell culture chambers were monitored after 19 DIV, showing (Fig. 1E) that both cortical and striatal neurons expressed NMDA receptors subtypes. At respectively DIV18 and DIV20, cortical and striatal primary neurons express both D1R and D2R detected by western blot analysis (Fig. 1F). Overall, these data indicate that the innervation of striatal cells by cortical neurons induce functional glutamatergic synapses. This led us to characterize the functional role that these synapses play in cortico-striatal dynamics.

### Global network analysis of calcium rhythms shows that cortical neurons drive synchronous oscillations in connected striatal neurons

**Figure 2:**
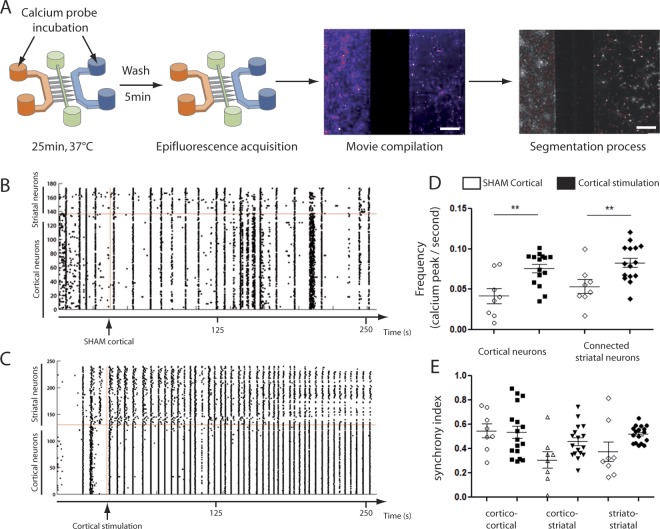
Low-resolution calcium imaging of reconstructed cortico-striatal networks. (**A**) Cortico-striatal networks were incubated with a calcium probe (Fluo4-AM) during 25 minutes at DIV19. Epifluorescence acquisitions at 5X, 500 ms time interval, are carried out to record the global network activity. Then, movies are analyzed and quantified (MATLAB plugins and routines, see Material and Methods). (**B**) Raster plot obtained showing spontaneous network activity of a representative network after SHAM stimulation (empty vehicle). (**C**) Raster plot showing network activity after cortical stimulation with a bicuculine/4-AP/nimodipine (cortical stimulation) cocktail. (**B**,**C**) Cortical stimulation is depicted by a vertical red line, while a horizontal red line marks the separation between cortical (below) and striatal (above) chambers. (**D**,**E**) Frequency and synchrony network quantification, higher index indicates stronger.

To characterize global network activity, calcium imaging was performed together with an analysis of burst frequency and synchrony in and between neuronal populations. Networks were loaded with a calcium probe, fluo4-AM, and recorded (time-lapse) at 5X magnification every 500 ms, to monitor both cortical and striatal activities (Fig. 2A). Automated image and statistical analysis processing (Fig. 2A) allowed for the extraction of raster plots (Fig. 1B,C) depicting the time-dependent and spatial occurrence of calcium bursts in all neurons recorded from the cortico-striatal network. Individual cells are depicted on the Y axis and time on the X-axis. To stabilize cortical neuron activity, a pharmacological cocktail was chosen, composed of bicuculline (50 µM), 4-aminopyridine (2.5 mM) and nimodipine (5 µM)^13^. Figure 2B,C show representative raster plots of cortical neurons, stimulated or not by the bicuculline/4AP/nimodipine cocktail. As shown in Fig. 2B, both cortical and striatal compartments from established networks (19DIV) exhibited synchronous activity (Suppl Fig. 1). Analysis of spontaneous calcium bursts rhythms showed that a 0.04 Hz (+/−0.013) average frequency in cortical neurons translated into 0.05 Hz (+/−0.011) frequency in striatal neurons. Cortico-cortical and striato-striatal synchrony were 50% and 40% respectively (Fig. 2D,E). Cortico-striatal synchrony was 40% (Fig. 2E), whereas unconnected striatal neurons did not show any sign of spontaneous oscillations^12^. This indicates that spontaneous bursting cortical neurons imprint their rhythms upon striatal neurons. Pharmacological treatment of cortical neurons with 50 µM bicuculline, 2.5 mM 4-AP and 5 µM nimodipine increased the frequency of oscillations in both cortical (t_(21)_ = 3.37, p = 0.003, unpaired t-test) and striatal (t_(21)_ = 2.89, p = 0.009, unpaired t-test) compartments (Fig. 2C–E) while record buffer perfusion (SHAM) did not. This analysis shows that spontaneous and evoked activity from all neurons in the cortico-striatal network can efficiently be probed in the microfluidic device, and indicates that striatal neurons were synchronously locked to cortical ones.

### GLUN2B receptors drive cortico-striatal synchrony

**Figure 3:**
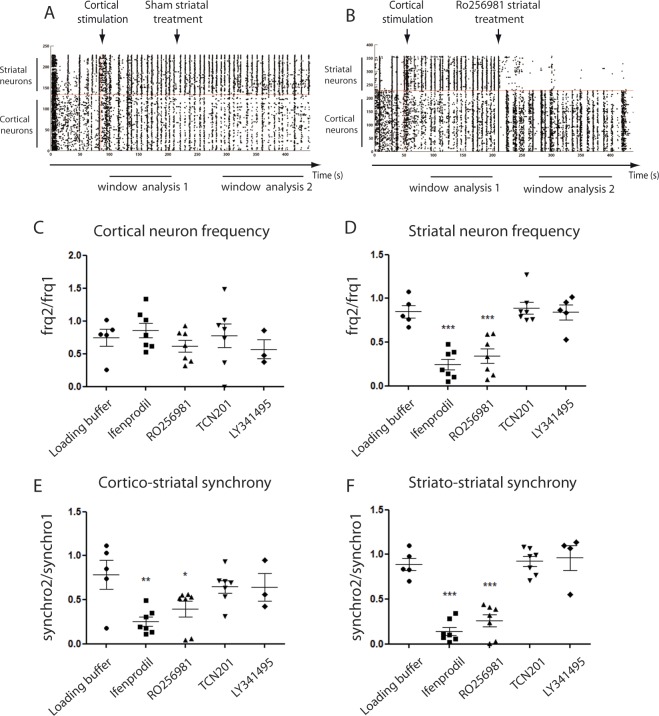
Characterization of glutamatergic transmission in cortico-striatal networks Calcium activities from DIV 19 cortico-striatal networks were recorded to characterize glutamatergic activity. (**A**,**B**) Representative raster plot obtained after cortical stimulation followed by a striatal treatment, SHAM (**A**) and RO256981 (**B**). Quantification of frequency (**C**,**D**) and synchrony (**E**,**F**) of cortico-striatal networks following the perfusion of specific glutamatergic transmission antagonists: ifenprodil, RO256981 for GluN-2B, TCN201 for GluN-2A and LY341495 for mGluR.

Glutamatergic transmission can affect cortico-striatal synchrony through ionotropic and metabotropic receptor pathways (see introduction). In order to identify their respective implication in the transmission of cortical oscillations to the striatal network, specific antagonists of glutamatergic receptors were perfused into the striatal chamber. Panels 3 A and 3B display representative raster plots from network activity after cortical stimulation (Fig. 3A) or cortical stimulation followed by striatal glutamatergic inhibition (Fig. 3B). Importantly, when cortico-striatal networks displayed spontaneous oscillations, perfusion of a pan-glutamatergic inhibitor into the striatal chamber disrupted striatal rhythms but did not disrupt cortical oscillations due to the fluidic isolation of the microfluidic device (F_(4,24)_ = 0.67; p = 0.62; one-way ANOVA, Fig. 3C). The impact of GluN2B receptors on striatal rhythms was assessed by perfusing 10 µM ifenprodil or RO256981 (Suppl Fig. 2B,C). As shown in Fig. 3D, both compounds induced a strong decrease in striatal frequency (one-way ANOVA followed by Newman-Keuls multiple comparison test: ifenprodil: q_(30)_ = 7.89, p < 0.05; RO256981: q_(30)_ = 6.63, p < 0.05, Fig. 3D). Cortico-striatal synchrony was significantly impacted by Ifenprodil and RO256981 (one-way ANOVA followed by Newman-Keuls multiple comparison test: ifenprodil: q_(28)_ = 5.41, p < 0.05; RO256981: q_(28)_ = 3.96, p < 0.05, Fig. 3E) and striato-striatal synchrony (one-way ANOVA followed by Newman-Keuls multiple comparison test: ifenprodil: q_(29)_ = 10.51, p < 0.05; RO256981: q_(29)_ = 8.83, p < 0.05, Fig. 3F). In contrast, perfusion of 10 µM TCN201 (Suppl Fig. 2D,E), 30 µM LY341495, antagonists of GluN2A and I, II and III mGluR respectively, had no significant effect (P values > 0.05) on the cortico-striatal network frequency and synchrony (Fig. 3C–F). This suggests that, in 19 DIV reconstructed cortico-striatal networks, cortico-striatal synchrony is mainly driven by receptors containing the GluN2B subunit.

### D2R dopamine receptors control glutamate-induced cortico-striatal synchrony

**Figure 4:**
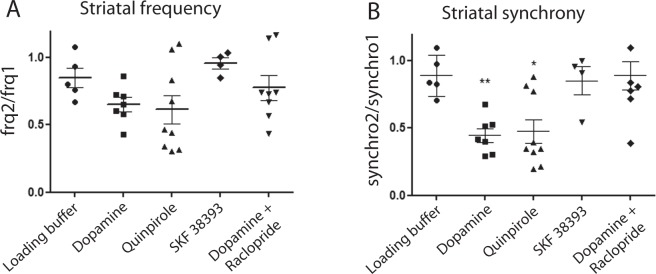
Characterization of dopaminergic modulation in cortico-striatal networks. Calcium activities from DIV 19 cortico-striatal networks were recorded to characterize the influence of dopamine agonist perfusion on cortico-striatal glutamatergic activity. Quantification of frequency (**A**) and synchrony (**B**) of striatal neurons following perfusion of dopaminergic agonists: (D1R and D2R), SKF38393 (D1R), quinpirole (D2R), or antagonists: raclopride (D2R).

A potential regulatory role of dopamine on evoked cortico-striatal activity was evaluated by acute perfusion of dopamine into the striatal chamber of our microfluidic device. Under these conditions, 10 µM dopamine triggered a significant diminution of striato-striatal synchrony and did not affect the frequency activity of cortico-striatal network (one-way ANOVA followed by Newman-Keuls multiple comparison test: q_(24)_ = 5.14, p < 0.05, Fig. 4A,B) (Suppl Fig. 3F,G). In order to identify which class of dopaminergic receptor may be implicated, 10 µM SKF 38393 or 10 µM quinpirole, D1R and D3/D2R agonists respectively, were perfused in the striatal chamber. As shown in Fig. 4A,B, SKF 38393 has no impact on striatal activity (one-way ANOVA followed by Newman-Keuls multiple comparison test: synchrony: q_(24)_ = 0.38, p > 0.05) whereas quinpirole induced a similar pattern of modifications (synchrony: q_(24)_ = 5.05, p < 0.05) as previously observed for dopamine. The role of D2R in striatal desynchronization was confirmed using raclopride, a selective D2/D3R antagonist. As shown in Fig. 4B, 10 µM raclopride abolished the effect of dopamine (one-way ANOVA followed by Newman-Keuls multiple comparison test, dopamine + raclopride vs dopamine + control: synchrony: q_(19)_ = 5.57, p < 0.05) and fully restored the synchronous oscillation behavior of the striatum, thus further confirming the specific involvement of D2R/D3R in attenuating the GluN2B cortically-driven striatal oscillations (Suppl Fig. 3H,I).

## Discussion

Neural circuit dynamics require elucidation in order to better understand complex syndromes such as Parkinson’s disease (PD) or schizophrenia, in which alterations of the cortico-striato-thalamo cortical loops have been extensively reported^14–16^. However, neural dynamics arise from complex assemblies of neurons in recurrent networks and re-entrant loops render those studies quite challenging^17,18^. This issue has been tackled by several experimental models, with different levels of physiological relevance, from the whole brain to brain slices or neuronal cultures. This latter method has been hampered by a lack of structural organization mimicking brain circuitry^19^. Recently, progress in cellular culture biotechnology has permitted the development of sophisticated *in vitro* devices for recording neuron circuit activity^19–22^. As exemplified herein, microfluidic modela of cortico-striatal networks are very instructive for reconstructing and analyzing oriented neuronal network development and probing the dynamics arising from a canonical re-entrant loop^12,23^. In addition, we show that the low resolution unbiased methodology to probe neuronal network activity can be exploited to study the regulatory influence of pharmacological agents upon glutamatergic and dopaminergic integration at cortico-striatal synapses. Fluo4-AM was chosen to record network activity since it is one of the most widely used non-ratiometric calcium probes for studying calcium imaging. It is easy to handle and it displays a high signal/noise ratio. A genetic calcium probe like GCaMP would be an alternative as this would open the possibility of expression in specific neuronal subtypes.

Overall, the present data show that rodent cortico-striatal networks reconstructed in a microfluidic environment are fully functional and exhibit characteristics close to those of physiological circuits. These networks reached a plateau of connectivity at 18–20 days of culture *in vitro*. The presence of VGLUT1 clusters on striatal spines, the pronounced complexity of striatal dendrites and the expression of NMDA receptors at the striatal post-synaptic density confirm the relevance of the present model for the study of oriented cortico-striatal networks. In addition, the spontaneous cortical oscillations observed by using the low-resolution calcium imaging technique indicate a strong synchrony between cortical neurons, as expected from a recurrent network^24,25^. These cortical oscillations were synchronously transmitted to striatal neurons, demonstrating the functionality of the reconstructed cortico-striatal feed-forward network. In contrast, asynchronous cortical activity did not propagate to striatal neurons. To stabilize the network activity, a specific pharmacological cocktail was perfused on cortical neurons. Several studies aimed to decipher synaptic induced pro-survival pathways in cortical or hippocampal neurons^13,26,27^. They showed that, at physiological Mg2 + levels, Nifedipine, another blocker of dihydropyridine calcium channels, does not abolish bicuculline/4-AP induced calcium oscillation but blunts oscillation frequency^28,29^. Based on this, the aim was to stabilize the intrinsic spontaneous rhythms of cortical cultures instead of using pharmacological conditions that trigger epileptic behavior in excitatory cultures. Hence, only highly synchronous cortical activity could induce calcium waves in striatal neurons, consistent with the known role of striatal cells as a detector of synchronous cortical activity^30^. Taken together, these data indicate that cultured cortico-striatal networks display characteristics similar to *in vivo* cortico-striatal networks, such as functional synapses with specific receptor expression, and a strong dependence of striatal neurons activation on correlated cortical activity.

*In vivo*, a developmental switch between GluN2A and GluN2B receptors is known to occur at around adolescence^31,32^ such that GluN2A receptors control synaptic activity in adulthood^33,34^. Interestingly, while both GluN2B *and* GluN2A subunits were expressed in the striatal compartment, glutamatergic transmission in striatal neurons appeared to be principally mediated by NMDA receptors composed of GluN1/GluN2B subunits^4,35^. Thus, even though synaptic connectivity reached a peak at days 18–20, it appears that cortico-striatal networks still presented an only partially mature phenotype. On the other hand, interactions between neurons and glia and the lack of chronic dopamine stimulation may influence receptor composition at the post-synaptic density with only NMDA receptors containing GluN2B subunits occurring in the striatal synaptic cleft. In line with this possibility, both astrocytes and dopamine have previously been shown to control the GluN2A/GluN2B receptor ratio at the synaptic cleft^36–38^

*In vivo*, dopamine concentrations can rapidly fluctuate and attain high levels: such phasic signaling underpins highly-regulated and dynamic roles other than those linked to the tonic, slow variations^39,40^. Although the temporal pattern of dopamine release cannot be controlled in the same manner as *in vivo*, acute perfusion of dopamine into the striatal chamber was carried out in order to mimic the effects of dopamine release. Importantly then, dopamine markedly modulated the influence of glutamate upon striatal networks, the major transmitter of cortico-basal loops. As described above, basal cortico-striatal synchrony was robust, and D2R activation delayed and desynchronized striatal responses to cortical bursts. Conversely, despite the fact that we could demonstrate the presence of D1R in our networks, D1R activation did not decrease cortico-striatal synchrony, suggesting a contrasting role for D1R. As D2R is recruited at lower dopamine concentrations than D1R, quinpirole perfusion might be considered as mimicking the effects of tonic dopamine release. These results are consistent with the notion that dopamine affects the activity of neuronal assemblies in the basal ganglia^41^, with D2R activation switching the network to an alternative pattern of activity^42^. Previous work reported an effect of SKF38393 in generation of calcium oscillations in 40% of MSN through a D1 receptor-mediated mechanism^43^. In this work, the authors used a 5 fold higher concentration of SKF38393 (50 µM) than in the present study. However, this is very high and risks cross talk to other sites. It is, then possible, that at lower concentration the amplitude of D1 receptor activation was too weak to be detected. The effect of SKF38393 at 10 µM may also be too transient to be captured using our experimental conditions as we used a relative low sampling rate of acquisition (pictures were taken every 500 ms). SKF38393 has been shown to regulate both Gs and Gq signaling in the striatum^44^, and thus may have an effect both on cAMP and intracellular calcium levels. Gs and Gq pathways may thus exert an opposite impact on the regulation of MSN calcium oscillations.

Interestingly, statistical analysis of striatal rhythms in microfluidic networks showed a 50% decrease of the synchrony index. This result suggests that only a subpopulation of striatal neurons were sensitive to quinpirole perfusion. While striatal neurons are known to express segregated D1R and D2R/D3R expression *in vivo*, due to unreliable immunofluorescence detection with commercially available antibodies, we were not able to assess *in vitro* D1R/D2R segregation. Yet, recent reports, using primary cultures derived from drd1a-tdTomato and drd2-GFP reporter transgenic mice, showed that segregated DR receptors occur *in vitro*^45^. It would be interesting to implement D1R and D2/D3R monitoring in our calcium rhythms assays, in order to accurately delineate the effect of dopamine on connected striatal neurons. Importantly, our results extend data from brain slices showing that cortical drive on striatal neurons, which is altered in animal models of Parkinson’s disease, is modulated by acute perfusion of dopamine^41,46,47^.

Taken together, the present data demonstrate that microfluidically-reconstructed neuronal networks acquire integrative properties that permit analysis of their complex patterns of activity and modulation with pharmacological agents. An interesting - albeit challenging - development of our novel platform would consist in the introduction of a third chamber containing dopaminergic neurons, but this would require the availability of pure dopaminergic primary cultures. ISPC-derived neurons might help solve this issue. Indeed, human iPSC-derived neurons from health subjects and patients could prove very insightful in modeling neurodevelopmental processes, as well as complex diseases such as neurodegenerative syndromes and psychiatric disorders. More generally, customized microfluidic devices are a versatile tool for evaluating the behavior of cortico-striatal and other complex cerebral circuits under both physiological and pathological conditions^12,23,48^. Further, microfluidic devices offer a novel tool for the initial screening and selection of novel pharmacological agents prior to the initiation of more onerous *in vivo* studies requiring considerable greater quantities of the chosen compounds.

To summarize, an innovative microfluidic system methodology coupled to real-time calcium imaging permitted the construction and dynamic analysis of physiologically-relevant cortico-striatal networks, and showed that their activity is principally driven by NMDA GluN2B subunit-containing receptors. Striatal neurons displayed VGLUT1 clustering on dendrites, spinogenesis and an increasingly complex dendritic tree until DIV19. Finally, D2R were found to monitor the glutamate-induced induction of cortico-striatal synchrony, although further work will be needed to more precisely characterize the control of network activity by additional classes of DA receptor and other mechanisms. Thus, neuronal networks reconstructed in microfluidic models display similar characteristics to *in vivo* circuits and permit the characterization of their dynamics and pharmacological modulation. This offers a framework for the evaluation of novel mechanisms for the therapeutic control of disorders involving disruption of cortico-striatal pathways.

## Methods

### Microfluidic chip production

The microfluidic channels were constituted of large channels (55 µm in height) for cell injection and thin channels (3 µm in height) for axon growth. To design the template, two layers of photoresist were used (SU82005 and SU82002). Silicon templates were replicated within another polyester resist (Dalbe). These two processes were previously detailed in^12^. The three compartmented chip (“3C”) was composed of: two rectangular macro-channels (length: 4000 µm; width: 500 µm; height: 55 µm) separated by arrays of asymmetrical microchannels (length 500 µm, 15 to 3 µm width, 3 µm height) interrupted by a third macro-channel inserted in the middle of the micro-channel “diode” array, as previously described (Peyrin J. M. *et al*.^12^). This third macro-channel acts as an intermediate compartment allowing controlling the flow of solution over the mid-portion of cortical axons^49^. The chip production for culture was done as following. PDMS (Sylgard 184) was mixed with a curing agent (9:1 ratio) and degassed under vacuum. The resulting preparation was poured onto a polyester resin replicate and reticulated at 70 °C for 2 hours. The elastomeric polymer print was detached and 2 reservoirs were punched for each macro-channel. The resulting piece was cleaned with isopropanol and dried. The polymer print and a glass coverslip were treated for 200 seconds in an air plasma generator (98% power, 0.6mBar, Diener ATTO) and bonded together. Chips were placed under UV for 15 minutes and then coated with a solution of poly-D-lysine (10 µg/ml) overnight and washed with PBS before cell seeding.

### Primary neuronal cultures

C57BL6N mice were purchased from Janvier (Le Genest Saint Isle, France) and bred in the animal facility at University Pierre et Marie Curie (IFR83). Animal care was conducted in accordance with standard ethical guidelines (U.S. National Institutes of Health publication no. 85–24, revised 1985, and European Committee Guidelines on the Care and Use of Laboratory Animals) and the local, IBPS and UPMC, ethics committee approved the experiments (in agreement with the standard ethical guidelines of the CNRS “Formation à l′Expérimentation Animale” and were approved by the “C2EA -05 Comité d’éthique en experimentation animale Charles Darwin”). Cortices and striata were micro-dissected from 7 to 10 E14 embryos of C57BL6N mice (Janvier, France). All steps of dissection were performed in cold PBS supplemented with 0.1% glucose. Dissected structures were digested with trypsin-EDTA for striata (Gibco) or papaïn for cortices (20U/ML in DMEM, Sigma). After tryspin or papaïn inactivation with FBS, structures were mechanically dissociated with a pipette in presence of DNAse. After several rounds of rinsing, cells were re-suspended in DMEM in a final density of 35 and 8 million cells/mL for cortices and striata, respectively. Cortical cells were then seeded in the somatic compartment and striatal cells in the distal compartment: 2 µl of the cell suspension was introduced into the upper reservoir and cells flowed into the chamber and adhered within 1–2 minutes. Cell culture medium was then added equally to the six reservoirs (60 µl/reservoir). Both neuronal cell types were grown in DMEM glutamax + streptomycin/penicillin (Gibco) + 5% FBS + N2 (Gibco) + B27 (Gibco). Microfluidic chips were placed in plastic Petri dishes containing H2O-EDTA to prevent evaporation and incubated at 37 °C in a humid 5% C02 atmosphere. The culture medium was renewed every 6 days.

### Lentiviral transduction in a microfluidic device

Cortico-striatal neurons were transduced at DIV4 in microfluidic devices. The volume of culture media was decreased to 20 µL in each reservoir. 3 µL of viral suspension, 10^8 viral particles per ml, was added to one of the reservoirs of each chamber. A 15 µL flow was induced in each chamber to perfuse the virus in the cell culture chamber. 6 hours after transduction, reservoirs were filled with conditioned media. Culture media was renewed at DIV6 as said in the primary neuronal culture part. The lentivirus used is a shRNA-CTL allowing the expression of a turboRFP as a transduction reporter (GE-Dharmacon, S13-005000-01).

### Cell extractions and Western Blot

For the detection of glutamatergic receptors, cells grown in microfluidic chips were homogenized in lysis buffer (0.5% Triton, 0.5% sodium dodecyl sulfate, 50 mM Tris HCL 150 mM NaCl) and protease inhibitor mixture (Invitrogen) on ice. Samples were analyzed with SDS-PAGE, followed by western blotting. For the detection of dopamine receptors, cells grown in 24-well plates were lysed directly in SDS buffer, heated to 95 °C for 3 min, and after cooling urea was added to samples until a final concentration of ~4 M. Samples were run on ultrathin (0.1 mm) gels^50^, and Western blots revealed with alkaline phosphatase-coupled secondary antibodies and NBT/BCIP reagent. The following antibodies were used for western blotting: GluN1 (05–432, Millipore, 1/1000), GluN2A (Millipore, 04–901, 1/500), GluN2B (Millipore, 05–920, 1/500), VGLUT1 (gift from Dr Salah El Mestikawy, IBPS CNRS UMR8246, Paris, France, 1/1000), D1R (Sigma, D2944-100, 1/500) and D2R (Millipore, ABN462, 1/500).

### Immunofluorescence

Cultures were fixed in 4% paraformaldehyde (PFA), 4% sucrose for 15 minutes at room temperature. Cells were then washed twice with PBS for 5 min and permeabilized for 45 min with 0.2% Triton X-100 and 1% BSA in PBS. Primary antibodies were then added and the samples incubated at 4 °C overnight in PBS. The samples were rinsed twice for 5 minutes with PBS and further incubated with the corresponding secondary antibodies for 2 hours at room temperature. The chips were then rinsed once with PBS and once with PBS + 0.1% sodium-azide. The following primary antibodies were used: VGLUT1 (gift from Dr. Salah El Mestikawy, IBPS CNRS UMR8246, Paris, France, 1/1000), MAP2 (M4403, Sigma, 1/500). Species-specific secondary antibodies coupled to Alexa 350, 488, 555 or 633 were used (1/500, Invitrogen) to visualize bound primary antibodies.

### Image acquisition

Images were acquired with an Axio-observer Z1 (Zeiss) fitted with a cooled CCD camera (CoolsnapHQ2, Roper Scientific). The microscope was controlled with Metamorph software (Molecular Imaging) and images were analyzed using ImageJ software (ImageJ, U. S. National Institutes of Health, Bethesda, Maryland, USA).

### Quantification of synaptic density

To quantify synaptic density, VGLUT1 cluster on striatal dendrites stained with MAP-2 were considered. With the mask of striatal cytoskeleton obtained as described in^23^, the VGLUT1 clusters are recovered by a “AND” mathematical operation. To eliminate small VGLUT1 cluster staining, the image is thresholded by the mean intensity of the image + 3*intensity standard deviation. After this thresholding, the number of clusters is enumerated and reported at the dendritic length found with ImageJ “skeletonize” plug-in of the mask of the striatal cytoskeleton.

### Live-Imaging and Calcium activity analysis

After 18–19 days *in vitro*, microfluidic devices were rinsed twice with recording buffer (NaCl 116 mM, KCl 5.4 mM, MgSO4 0.8 mM, CaCl2 1.8 mM, NaH2PO4 1.3 mM, Glucose 10 mM, Hepes 10 mM, bicarbonate 25 mM, glycine 10 µM dissolved in sterile water). Cells were then incubated for 25 min at 37 °C with the loading buffer (NaCl 116 mM, KCl 5.4 mM, MgSO4 0.8 mM, CaCl2 1.8 mM, NaH2PO4 1.3 mM, Glucose 10 mM, Hepes 10 mM, bicarbonate 25 mM, glycine 10 µM) supplemented with 2 µM fluo4 and 20% pluronic acid (1/1000), dissolved in sterile water. After 2 rinses with recording buffer, cells were recorded at room temperature under the microscope. Each frame was acquired every 500 ms for 10 minutes. Segmentation of movies was performed under MATLAB environment using Caltracer version 2.5 developed by the Yuste laboratory. Only calcium transients elicited by neurons were considered by removing calcium transients generated by glial cells. Astrocytes show large somatic compartments compared to neurons, and display slow calcium wave activity, with a lower frequency compared to neuronal calcium activity. The area of ROIs was thus adjusted in order to exclude astrocytes based on morphology (area < 200 µm^2^), and remaining glial activity was removed based on peak frequency and intensity. Active neurons were then semi-automatically identified using a custom-made MATLAB routine available upon request. Each neuron was numbered and assigned a two-dimensional coordinate to locate it in one of the two chambers. Neuronal calcium transients were analyzed as continuous values (fluorescence intensity at an acquisition rate of 2 Hz) or as point processes, according to the following detection of peak activity. Calcium transients were detrended to remove slow fluctuations in the signal, then a peak was detected at the maximal intensity each time the signal crossed a threshold, set at the mean + standard deviation of the detrended signal. These peaks in calcium activity were used to construct binary matrices (1 if neuron is active, 0 otherwise) that can be depicted as raster plots (each row denotes the number of an active neuron, columns represent the time frame) with dots representing activity. Average firing frequency was computed from the total number of peaks over the duration of a given treatment (e.g. buffer control, cortical stimulation) when the network response reached steady state, i.e. after removing the transitory response, which may have reflected the mechanical effects of solution perfusion in the microfluidic device. The mean synchrony was averaged over pairwise synchronies (computed with Kreuz *et al*.^51^ spike-synchronization index), over the same periods of time used for firing frequency. Briefly, we measured pairwise synchronies among all pairs of neurons, by quantifying the quasi-simultaneous appearances of peaks in the calcium signal. Calcium peaks were considered as quasi-simultaneous if they fell in a coincidence window adapted to each neuron’s peak rate^51^. Hence this synchrony index is parameter and scale free. Network synchrony was taken as the average synchrony index over time and neurons.

### Live pharmacological treatment

All chemicals were prepared as concentrated solutions according to the recommendations of the different manufacturers. Compounds were aliquoted in Eppendorf tubes and used once, to avoid repeated freezing/thawing processes. Aliquots were stored at −80 °C for no longer than two months. Care was taken to protect photosensitive molecules from light by wrapping the test tubes in aluminum foil. Drugs were extemporaneously diluted at their respective final concentration in loading buffer. For pharmacological treatments, the medium was removed from the cortical chambers and/or striatal chamber and was replaced by recording buffer containing or not (Sham) pharmacological drugs. The following concentrations were used: to stabilize the cortical neurons activity, microfluidic cortical compartment was subjected to a 50 µM bicuculline (Tocris, 0131), 2.5 mM 4-AP (Tocris, 0940) and 5 µM nimodipine (Tocris, 0600) cocktail and recorded for 3 minutes, after which specific glutamatergic or dopaminergic receptor antagonists were perfused in the striatal compartment. TCN201 (Tocris, 4154), a GluN2A antagonist^52^, Ifenprodil (Tocris, 2892) and RO256981 (Tocris, 1594), GluN2B antagonists^53,54^ were used at 10 µM and LY341495 (Tocris, 1209), mGluR antagonist, were used at 30 µM^55^. SKF 38393, a D1R agonist (10 µM; Tocris, 0922), Quinpirole, a D2/D3R agonist (10 µM, Tocris, 1061), Raclopride, a D2/D3R antagonist (1 µM, Tocris, 1810).

## Electronic supplementary material

Supp Fig1 Record A

Supp Fig2 Record B

Supp Fig2 Record C

Supp Fig2 Record D

Supp Fig2 Record E

Supp Fig3 Record F

Supp Fig3 Record G

Supp Fig3 Record H

Supp Fig3 Record I

Supplementary Figures and Legends
