# Supplementary material for: Glutamatergic and dopaminergic modulation of cortico-striatal circuits probed by dynamic calcium imaging of networks reconstructed in microfluidic chips
Source: Sci Rep. 2018 Nov 29;8:17461. doi: 10.1038/s41598-018-35802-9 (PMC6265304; doi:10.1038/s41598-018-35802-9)
Supplement: Supplementary file 10 — Supplementary Figures and Legends [file 41598_2018_35802_MOESM10_ESM.pdf]

Glutamatergic and dopaminergic modulation of cortico-striatal circuits probed by dynamic calcium imaging of networks reconstructed in microfluidic chips

Authors and affiliations: Benjamin Lassus[1,3], Jérémie Naudé[2,3], Philippe Faure[2,3], Denis Guedin[4], von Boxberg Y[2,3], Clotilde Mannoury la Cour[5], Mark J Millan[5], and Jean-Michel Peyrin[2,3*]

Supplementary information

Supplementary figure 1:

Recording A: Calcium activity from DIV 19 cortico-striatal networks after the stimulation of cortical neurons with 50µM bicuculine, 2.5mM 4-AP, and 5µM nimodipine cocktail. On this recording, only one part of the device is showed. The cortical compartment is on the left, the striatal compartment on the right, and the array of microchannels linking the two compartments containing cortical axons is in the middle. Cortical and striatal compartments exhibit oscillatory, synchronous calcium activity. Video is 15 fps. Recording A duration: 150 seconds. Video resolution: 1.175.2 µm x 405.6 µm.

Supplementary figure 2:

Recordings B and C: Calcium activities from DIV 19 cortico-striatal networks. Recordings **B** and **C** show the activity of the connected striatal compartment before (recording **B**) and after (recording **C**) the perfusion of a GluN2B antagonist (RO256981) in the striatal chamber. On the recording **B**, the synchronous activity of striatal neurons is shown before the perfusion of RO256981, whereas on the recording **C** the activity of striatal neurons is abolished after the perfusion of RO256981. Videos are 15 fps. Recording B duration: 200 seconds. Recording C duration: 150 seconds. Resolution for each video: 499.2 µm x 322.4 µm.

Recordings D and E: Calcium activities from DIV 19 cortico-striatal networks. Recordings **D** and **E** show the activity of the connected striatal compartment before (recording **D**) and after (recording **E**) the perfusion of a GluN2A antagonist (TCN201) in the striatal chamber. On the recording **D**, the synchronous activity of striatal

neurons is shown before the perfusion of TCN201. On the recording **E,** TCN201 does not exert any noticeable effect on the activity of striatal neurons. Videos are 15 fps. Recording D duration: 475 seconds. Recording E duration: 150 seconds. Resolution for each video: 499.2 µm x 322.4 µm.

Supplementary figure 3:

Recordings F and G: Calcium activities from DIV 19 cortico-striatal networks. Recordings **F** and **G** show the activity of the connected striatal compartment before (recording **F**) and after (recording **G**) the perfusion of dopamine in the striatal chamber. On the recording **F**, the synchronous activity of striatal neurons is shown before the perfusion of dopamine whereas on the record **G** asynchronous striatal activity is triggered by the perfusion of dopamine. Videos are 15 fps. Recording F duration: 110 seconds. Recording G duration: 195 seconds. Resolution for each videos: 499.2 µm x 322.4 µm.

Recordings H and I: Calcium activities from DIV 19 cortico-striatal networks. Recordings **H** and **I** show the activity of the connected striatal compartment before (recordings **H**) and after (recordings **I**) the perfusion of dopamine and of a D2/D3R antagonist (raclopride) in the striatal chamber. On the recording **H**, the synchronous activity of striatal neurons is shown before the perfusion of dopamine. On the record **I**, no asynchronous activity is detected after the perfusion of dopamine and raclopride, stressing the role of D2 and D3R in the regulation of striatal activity. Videos are 15 fps. Recording H duration: 175 seconds. Recording I duration: 225 seconds. Resolution for each video: 499.2 µm x 322.4 µm.

Supplementary figure 4:

Full scan of western blot analysis presented as cropped gels in Figure 1. Major proteins linked to ionotropic glutamatergic transmission from microfluidic cortico-striatal network. **A.** VGLUT1 **B.** GluN2B **C.** GluN2A **D.** GluN1**.** **E.** D1 and D2 Dopamine receptors western blot full scan, from respectively DIV18 cortical neurons and DIV 20 striatal neurons grown in conventional P24 cell culture wells.

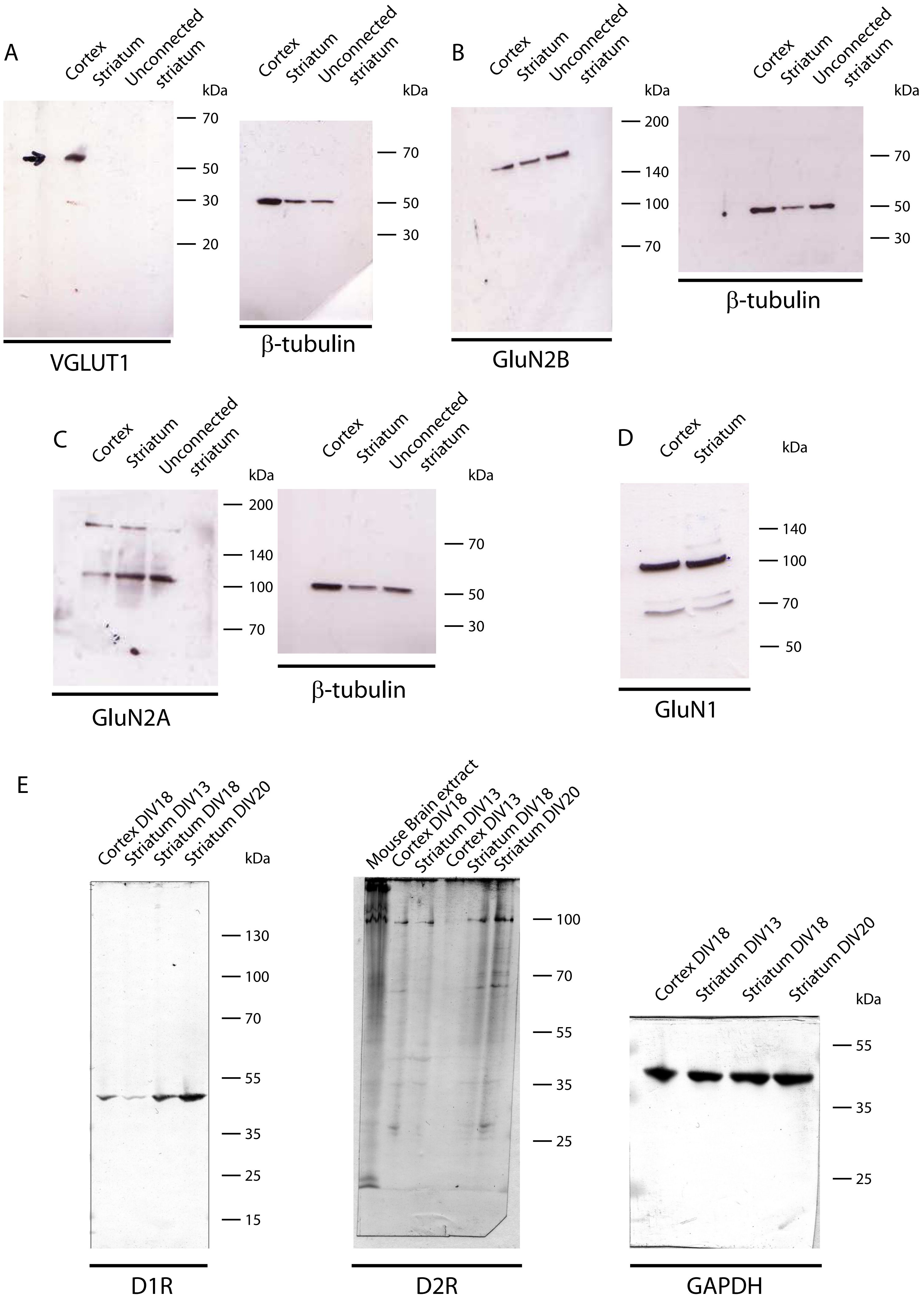
A
Cortex
Striatum
Unconnected striatum
kDa
70
50
30
20
VGLUT1
Cortex
Striatum
Unconnected striatum
kDa
70
50
30
β-tubulin
B
Cortex
Striatum
Unconnected striatum
kDa
200
140
100
70
GluN2B
Cortex
Striatum
Unconnected striatum
kDa
70
50
30
β-tubulin
C
Cortex
Striatum
Unconnected striatum
kDa
200
140
100
70
GluN2A
Cortex
Striatum
Unconnected striatum
kDa
70
50
30
β-tubulin
D
Cortex
Striatum
kDa
140
100
70
50
GluN1
E
Cortex DIV18
Striatum DIV13
Striatum DIV18
Striatum DIV20
kDa
130
100
70
55
35
25
15
D1R
Mouse Brain extract
Cortex DIV18
Striatum DIV13
Cortex DIV13
Striatum DIV18
Striatum DIV20
100
70
55
35
25
D2R
Cortex DIV18
Striatum DIV13
Striatum DIV18
Striatum DIV20
kDa
55
35
25
GAPDH
